# Air Quality and Respiratory Health among Adolescents from the United Arab Emirates

**DOI:** 10.1155/2015/284595

**Published:** 2015-05-17

**Authors:** Caroline Barakat-Haddad, Sheng Zhang, Ayesha Siddiqua, Rania Dghaim

**Affiliations:** ^1^Faculty of Health Sciences, University of Ontario Institute of Technology, 2000 Simcoe Street North, Oshawa, ON, Canada L1H 7K4; ^2^University of Toronto Scarborough, 1265 Military Trail, Toronto, ON, Canada M1C 1A4; ^3^Department of Clinical Epidemiology and Biostatistics, McMaster University, 1280 Main Street West, Hamilton, ON, Canada L8S 4L8; ^4^Department of Natural Science and Public Health, College of Sustainability Sciences and Humanities, Zayed University, Dubai, UAE

## Abstract

*Purpose*. To examine the role of air quality in relation to chronic bronchitis, emphysema, asthma, wheeze, and dry cough among adolescents from the United Arab Emirates (UAE). *Methods*. A survey was administered on 6,363 adolescents from 9 UAE regions. Data consists of demographic, socioeconomic, residential, and behavioural variables, such as location of residence, residing near industry/gas stations/dumpsites/construction sites, residing near overhead power line/plants, exposure to tobacco, residential exposure, ethnicity, concern over air pollution, smoking, and purposely smelling gasoline fumes/glue/correctors/car exhaust/burning black ants. Logistic regression modeling was used to determine significant predictors of respiratory health. *Results*. Asthma prevalence was 12.3%, followed by chronic bronchitis (1.8%) and emphysema (0.5%). Overall 12.2% reported wheeze and 34.8% reported a dry nocturnal cough in the past year. Multivariate analyses suggest that sex is a significant predictor of asthma and dry cough. Exposure to tobacco and arts/crafts/ceramics/stain is significant predictor of respiratory health. Tobacco smoking and purposely smelling gasoline fumes/glue/correctors/car exhaust/burning black ants are significant predictors of wheeze and dry cough. *Conclusions*. This study suggests that exposure to air quality and behavioral factors such as smoking and purposely smelling gasoline fumes, glue, correctors, car exhaust, or burning black ants are significant predictors of respiratory health among UAE adolescents.

## 1. Introduction

Recently, the World Health Organization identified indoor and outdoor air pollution as the “world's largest single environmental health risk” [1]. The report focused on the number of deaths attributed to air pollution and acknowledged air pollution's role in the development of respiratory diseases. Millions of people suffer from preventable chronic respiratory conditions worldwide [2, 3]. Chronic respiratory conditions represent a major public health challenge in both developing and industrialized countries due to their frequency and economic impact, through increased health care cost and lost disability adjusted life years [4].

Major preventable chronic respiratory conditions are chronic obstructive pulmonary disease (COPD), asthma, and occupational lung diseases [5]. COPD is generally characterized by the presence of chronic bronchitis or emphysema that can lead to airway obstruction [6]. Chronic bronchitis is a highly prevalent respiratory disorder, and emphysema occurs when the air sacs in the lungs are gradually destroyed [7]. Although subcutaneous emphysema and chronic bronchitis can result from repeated infections or injury in childhood or early adulthood [8–10], asthma is the most prevalent chronic respiratory condition of childhood [11]. Respiratory symptoms are generally used to suggest the presence of acute or chronic respiratory conditions. For example, wheeze can be a symptom of asthma and COPD [12]. Dry cough can be a symptom of many conditions as well, such as asthma, COPD, pulmonary fibrosis, and rhinosinusitis [13]. In regions where diagnoses with respiratory conditions are underestimated, respiratory symptoms can serve as possible proxies.

Literature has documented the impact of several risk factors on respiratory health. For example, chronic bronchitis is associated with cigarette smoking, passive smoking, air pollution, and exposure to dust or gas [14–16]. Risk factors for developing emphysema include cigarette smoking, exposure to poorly vented cooking and heating fires, trauma, and repeated infections [8–10, 17, 18]. Air pollution, including indoor pollution and traffic exposure, and living in urban setting are associated with an increased risk of asthma in children [11, 19]. There is also strong evidence linking parental smoking with asthma in children [20, 21].

**Figure 1:**
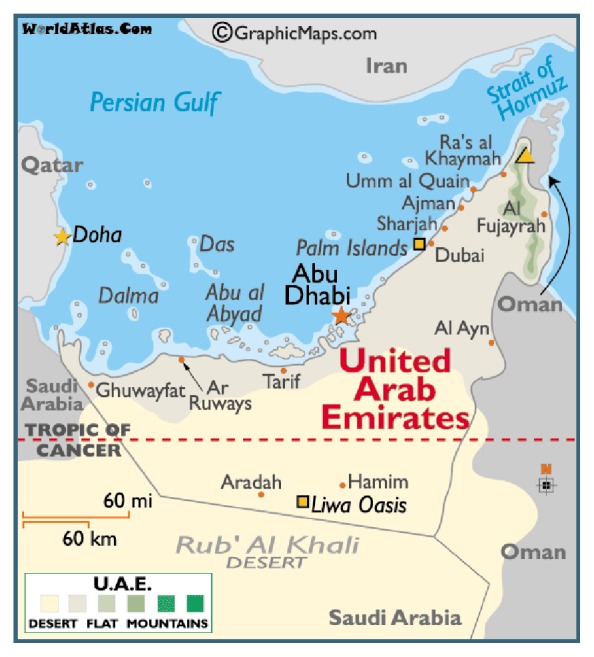
Map of the United Arab Emirates (UAE). Source: http://www.worldatlas.com/webimage/countrys/me.htm.

The United Arab Emirates (UAE) is a federation of seven emirates situated in the Arabian Gulf peninsula (Figure 1). The emirate of Abu Dhabi consists of three geographical regions, Abu Dhabi city, Al Ain, and the western region. While the UAE is dependent on oil production [22], regions are diverse in their dependence on agriculture, industry, or tourism. In the last few decades, the UAE has seen tremendous growth which has improved the living conditions of its people; however, it may have created conditions that lead to health risks [22–29], such as an alarming increase in the number of cars, large developments, an increase in industrial processes, and practices such as regular fogging. Research documents high prevalence of asthma and cardiovascular diseases, which are the leading cause of overall death in the UAE [30]. Indoor air quality has been linked to asthma and wheeze in a recent 2012 study that is reported as “one of the first assessments of the relationship between indoor air pollutant exposure and health outcomes in a region where environmental health studies are rare” [31]. Indeed, health research in general in the UAE is relatively scarce, and environmental health research is in its infancy.

To our knowledge, no studies have comprehensively examined the respiratory health profile among adolescents and the possible link with air quality in the UAE. This paper addresses two main objectives: (i) determining the prevalence of chronic bronchitis, emphysema, asthma, wheeze, and dry cough among UAE adolescents and (ii) exploring predictors of these health outcomes in the UAE adolescent population.

## 2. Methods

### 2.1. Data Source

This study utilizes data from the National Study of Population Health in the UAE (NSPHUAE) (2007–2009) research program that was undertaken in collaboration with the UAE Ministry of Education. A cross-sectional survey was developed and administered on 6,363 adolescents, aged 13 to 20 years, who attend public and private schools in the seven Emirates of the federation, including 9 educational zones. The researchers held training workshops for social workers, employed by the UAE Ministry of Education. The social workers administered the cross-sectional survey on students from three randomly selected classes from each participating school (one class from each of grades 10, 11, and 12). The first component of the survey was administered on the adolescent participants during a spare period in a classroom setting. Social workers collected the completed survey at the end of the defined one-hour period. The first component collected data on smoking behaviours, physical activity, self-reported medical diagnoses, symptoms in relation to respiratory health, and demographic and socioeconomic data. The second component of the survey was sent home with the student overnight, in order for the student to seek parental assistance in completing it. When the student participants returned it the following day, a participant identification code allowed it to be matched to the first component. The second component of the survey collected data related to the number of previous residences and locations, as well as residential and neighborhood characteristics (see Barakat-Haddad 2013 [32] for more detailed information).

### 2.2. Outcome Variables

In addressing the first objective, five items from the questionnaire were used to determine outcome measures, including three items adopted from the International Study of Asthma and Allergies in Children (ISAAC) [33]. Participants were asked if a doctor or health professional ever told them that they have chronic bronchitis or emphysema. A section related to the ISAAC questionnaire asked participants if they ever had asthma, if they experienced wheezing or whistling in the chest in the last 12 months, and if they had a dry cough at night, apart from a cough associated with a cold or chest infection in the last 12 months (see appendix).

### 2.3. Explanatory Variables

In relation to exposure to outdoor air quality, participants were classified based on their residential location in relation to the 9 geographic regions. In addition, responses to two items were used as proxies of exposure; participants were asked whether they reside near industrial plants, gas stations, dumpsites, or construction sites and near overhead power lines or plants. Exposure to indoor air quality was assessed using responses to items that asked how often the participant is exposed to tobacco at home or with friends (daily/occasionally/not at all), whether the property of residence was built prior to 1988 (this variable was used as proxy for exposure to lead in residential paint and other construction materials that are known to impact health), whether the residence has air conditioning, how often the residence is maintained in terms of cooling equipment, air filtration, and/or air duct cleaning (yearly/as needed/rarely), the type of flooring in the main living areas (TV room, living rooms, and bedrooms) of the residence (wall-to-wall carpet/ceramic tiles/wooden floors/heavy rugs/other), the type of cooking method used (electricity/gas/microwave/other), whether the residence feels humid, whether there are pets that live in the residence, whether there are pests (e.g., cockroaches, rodents) seen in the residence (yes/sometimes/no), whether the residence is subject to use of pesticides or insecticides (yes/sometimes/no), and whether anyone in the participants' household does arts, crafts, ceramics, stained glass work, or similar hobbies on a regular basis.

Data related to the psychological and behavioral profiles of participants included whether participants were concerned about air pollution in their neighborhood, whether they ever smoked cigarettes or any form of tobacco such as shisha or midwakh, and whether they smoked cigarette, shisha, midwakh, or other tobacco products occasionally or daily in the past 30 days. Participants were categorized as current smokers if they reported occasional or daily use of at least one form of tobacco in the past 30 days. This categorization is consistent with WHO guidelines [34]. In relation to substance abuse, participants were asked if they ever used illegal drugs such as marijuana, hashish, or cocaine. In relation to unconventional drug use, respondents were asked whether they ever purposely smelled gasoline fumes, glue, correctors, car exhaust, or burning black ants. These “other” forms of substance use are common knowledge among the UAE adolescent population. A series of questions asked participants whether they participated in any of a number of activities in the past 12 months.

Data related to the participants' demographic and socioeconomic profiles include sex, type of school attended, whether the participant was born in the UAE (indicative of long-term exposure to the UAE environment), ethnicity, monthly family income, parents' education level, residential property tenure, the number of individuals who reside in household, and the number of bedrooms in residence. A residential crowding variable was calculated using the ratio for the number of individuals who reside in household over the number of bedrooms in residence. Ethnicity was classified on the basis of similar cultures, traditions, ancestral linkages, or geographical origins.

### 2.4. Statistical Analysis

Data were analyzed using SPSSv20. Descriptive statistics were calculated for outcome and explanatory variables. Chi-square statistics was used to compare participants who reported health outcomes to their counterparts.

All independent variables that were significant in the bivariate analyses were entered into logistic regression models to identify significant predictors of respiratory conditions and symptoms. For each of the modeled outcomes, entry of independent variables was conducted using a significance level of *p* ≤ 0.05. In addition, given that our outcome measures are self-reported and that multiple studies report that subcutaneous or congenital emphysema can only occur in earlier ages due to trauma, infections, or injury [8–10], logistic regression modeling for emphysema includes other self-reported respiratory health outcomes as explanatory variables.

## 3. Results

This paper is based on responses from 6,363 adolescents aged between 13 and 20 years who reside in the UAE. Detailed information in relation to the number of schools sampled and response rate has been published elsewhere [32]. Overall, 50% of participants in this study were of local national origin and 45% of participants were male. The mean age of the study sample was 16 years. Tables 1 and 2 summarize the sociodemographic, environmental, and behavioural profiles of the study participants. The majorities of participants attend public schools (61%) and reside in the emirates of Abu Dhabi (47%) or Sharjah (18%). A high proportion of participants report residing near industrial plants/gas stations/dumpsites/construction sites (15%), residing near overhead power lines or plants (11%), exposure to tobacco at home or with friends (40%), residential pests (e.g., cockroaches, rodents) (60%), use of pesticides or insecticides (72%), and exposure to arts/crafts ceramics/stained glass work/similar hobbies (19%). Only 28% of participants report yearly residential maintenance of air filtration duct cleaning and over 68% report extreme, moderate, or slight concern about air pollution in their neighborhood. Overall, 14% of participants are current smokers and 29% have purposely smelled gasoline fumes, glue, correctors, car exhaust, or burning black ants.

Overall, 115 participants (1.8%) reported diagnosis with chronic bronchitis compared to 28 with emphysema (0.5%) and 776 with asthma (12.3%), 740 reported wheeze (12.2%), and 2,125 reported dry cough in the past year (34.8%) (Table 3). For all respiratory conditions and symptoms, the prevalence was higher in males than females, except for the prevalence of dry cough, where the prevalence was higher in females. For at least one respiratory condition or symptom, significant differences were found in relation to sex, school attended, birth in UAE, ethnicity, parents' education level, and residential property tenure (Table 3), as well as all three proxies of outdoor air quality, 7 proxies of indoor air quality, concern over air pollution, and all behavioral variables (Table 4).

Multivariate regression modelling reveals that geographical region and residing near industrial plant, gas station, dumpsite, or construction sites are predictors of asthma (Table 5). Exposure to tobacco is a predictor of asthma and respiratory symptoms. Residential humidity is a predictor of wheeze whereas exposure to residential pests (e.g., cockroaches, rodents) is a predictor of dry nocturnal cough. Having someone in the household who is regularly involved in arts/crafts/ceramics/stained glass work/similar hobby work is a predictor of both chronic bronchitis and emphysema. Concern over air pollution, having ever smoked, and purposely smelling gasoline fumes/glue/corrector/car exhaust/burning black ants are significant predictors of wheeze and dry cough. Ethnicity is a significant predictor of chronic bronchitis and asthma. Finally, diagnosis with chronic bronchitis and reporting wheeze are significant predictors of emphysema.

## 4. Discussion

Population-based health surveillance data is essential for healthcare and public health planning. Preventable chronic respiratory conditions cause premature deaths and result in decreased work productivity and negative economic impact on families, communities, and the society [5, 35]. Furthermore, the presence of respiratory conditions such as chronic bronchitis and asthma is associated with higher risk of other disorders, which can lead to increase in the overall disease burden. Our study shows that chronic bronchitis, emphysema, asthma, wheeze, and dry cough are quite prevalent among UAE adolescents and should hence be prioritized in the UAE national public health agenda. Specifically, the prevalence proportions of chronic bronchitis and emphysema obtained in our sample are comparable to those in the US for the age category 18–44 years [36]; in addition, results of the ISAAC questionnaire in countries neighboring the UAE suggest that the prevalence of asthma among adolescents is between 5 and 10%, of wheeze between 6 and 11%, and of dry nocturnal cough below 20% [37].

Adolescents living in Abu Dhabi city and the western region of Abu Dhabi and those who reside in proximity to industrial plants/gas station/dumpsite or construction sites are more likely to have asthma. This is not surprising given that air quality has been linked to asthma, and Abu Dhabi and towns in the UAE western region such as Al Ruwais primarily generate their income through oil and gas industries, which are contributors of air pollution. It is encouraging to note that recently the Environment Agency Abu Dhabi began preparing an air-quality strategy to implement pollution limits for industries in the capital [38].

Our findings suggest that adolescents who are exposed to tobacco at home or with friends are more likely to report diagnosed asthma, wheeze, and dry cough. This is not surprising as exposure to environmental tobacco is a well-recognized risk factor of asthma and respiratory symptoms [20, 39, 40]. This highlights the importance of supporting antitobacco messages and education campaigns targeting families and adolescents regarding the risks of tobacco use [41]. These strategies can be useful in reducing the burden of respiratory conditions in the UAE. Consistent with literature, our results suggest that adolescents who report residential humidity are also more likely to report wheeze [42]. Our findings also indicate that adolescents who have pests (e.g., cockroaches, rodents) in their residence are more likely to report dry cough. While the link between pests, a biological pollutant, and cough remains to be established, there is evidence suggesting that pests can trigger asthma [43]. Our study suggests that paraoccupational exposure to arts/crafts/ceramics/stained glass work/similar hobbies may be linked to diagnosis with chronic bronchitis and emphysema among UAE adolescents. This is not surprising, given that inhalation and exposure to dust of silica—which is a component of traditional ceramics and glass—can increase the risk of developing respiratory conditions [44, 45].

Although our results suggest that male adolescents are more likely to have diagnosed asthma, females are more likely to report dry cough which may be an indicator of undiagnosed respiratory conditions. Interestingly, research suggests that although males are more likely to have asthma in childhood and adolescence, there may be a reversal of this sex ratio in the long-term [46, 47]. We also found variations in chronic bronchitis and asthma diagnosis in terms of ethnicity. Adolescents from the UAE, GCC, Arab/Middle East, Arab/North Africa, and western countries are more likely to have diagnosed chronic bronchitis, while adolescents from the UAE, GCC, Arab/North Africa, and western countries are more likely to report asthma. Smoking behaviour is more prevalent among Arab populations than those from South East Asia [48, 49]. This may be related to the increased burden of respiratory conditions among the Arab subpopulations.

We found that adolescents who are concerned about air pollution in their neighbourhood are more likely to report wheeze and dry cough. Consistent with literature findings, our results suggest that smoking and ever purposely smelling gasoline fumes, glue, correctors, car exhaust, or burning black ants are predictors of wheeze and dry cough [10, 50–52]. Given the high availability of tobacco products, the social acceptance of smoking in the UAE, and the high prevalence of tobacco use among UAE adolescents [40], local strategies to curb this behaviour are commended to reduce the burden of respiratory conditions among UAE adolescents. In the UAE, although there is zero tolerance for illegal drug use and harsh judicial penalties, adolescents often choose to smoke burning ants, which are high in formic acid, as it is a legal alternative to smoking marijuana [53]. This highlights the importance of developing education campaigns to inform adolescents regarding the health threats of using unconventional drugs such as purposely smelling gasoline fumes, glue, correctors, car exhaust, or burning black ants.

This study was subject to several limitations. Data are self-reported and may have been subject to response bias. Specifically, the health outcomes derived did not account for the type of health outcomes; for example, we did not request information on the type of emphysema reported. This is particularly relevant given that emphysema tends to be common among older smokers but is not usually observed among adolescents. However, given that multiple studies report that subcutaneous or congenital emphysema can occur in earlier ages due to trauma, infections, or injury, we opted for exploring the link between emphysema and explanatory variables while including respiratory outcomes in the latter. Sampling led to lower representations of adolescents who attend private schools in Dubai and among males who reside in the UAQ. This is relevant as the population of Dubai consists of a large proportion of expatriates; hence, results related to the expatriate population in Dubai are likely to be biased. Importantly, our study does not account for the actual distances of participants' residences from industrial plants, gas stations, dumpsites, construction sites, overhead power lines, or plants, which could modify the relationship between exposure to poor air quality and health outcomes. Furthermore, response to the self-administered survey may have been influenced by the presence of social workers, with the possibility of underreporting of tobacco use among females given social norms. Despite these limitations, this study contributed to knowledge of a detailed profile and environmental predictors of respiratory conditions and symptoms among UAE adolescents that is crucial for public health planning.

## Figures and Tables

**Table 1:** Sociodemographic profile of the study participants (*n* = 6,363).

Variable	Classification	Male *n* (%)	Female *n* (%)	Overall *n* (%)
Sex		2791 (44.7)	3458 (55.3)	

School	Public	1975 (64.3)	1433 (58.6)	3820 (61.1)
	Private	996 (35.7)	2025 (41.4)	2429 (38.9)

Born in UAE	Yes	1216 (44.5)	1798 (52.6)	3014 (49.3)

Ethnicity^‡^	UAE	1216 (44.5)	1798 (52.6)	3014 (49.0)
	GCC	227 (8.3)	125 (3.7)	352 (5.7)
	Arab/Middle East	517 (18.9)	453 (13.3)	970 (15.8)
	Arab/Africa	327 (12.0)	291 (8.5)	618 (10.1)
	South East Asia	378 (13.8)	659 (19.3)	1037 (16.9)
	Western	30 (1.1)	51 (1.5)	81 (1.3)
	None/other	36 (1.3)	41 (1.2)	77 (1.3)

Father graduated from high school	Yes	1259 (56.9)	1649 (58.7)	2908 (57.8)

Mother graduated from high school	Yes	1069 (48.5)	1409 (49.5)	2478 (49.0)

Monthly household income (AED)	<2 K	87 (5.0)	74 (3.5)	161 (4.2)
	>2 and ≤5 K	441 (25.2)	445 (21.3)	886 (23.1)
	>5 and ≤8 K	304 (17.4)	369 (17.7)	672 (17.5)
	>8 and ≤10 K	211 (12.1)	258 (12.4)	469 (12.2)
	>10 and ≤12 K	160 (9.2)	188 (9.0)	348 (9.1)
	>12 and ≤15 K	125 (7.2)	188 (9.0)	313 (8.2)
	>15 and ≤20 K	114 (6.5)	171 (8.2)	285 (7.4)
	>20 K	106 (17.5)	395 (18.3)	701 (18.3)

Residential property tenure	Own	1140 (48.8)	1504 (52.5)	2687 (51.0)
	Rent	1195 (51.2)	1359 (47.5)	2578 (49.0)

Residential crowding	Yes	794 (61.3)	1249 (60.0)	2043 (60.5)

^‡^UAE = local; GCC = Kuwait, Kingdom of Saudi Arabia, Oman, Qatar, Bahrain, and Yemen; Arab/Middle East = Lebanon, Syria, Jordan, Palestine, and Iraq; Arab/Africa = Egypt, Tunisia, Morocco, Algeria, Libya, Sudan, and Somalia; South East Asia = India, Pakistan, Bangladesh, Sri Lanka, Philippines, and Indonesia; Western = Europe, USA, Australia, and Canada; no nationality and others = all other nationalities.

**Table 2:** Environmental exposure and behavioural profiles of study participants (*n* = 6,363).

Variable	Classification	Male *n* (%)	Female *n* (%)	Overall sample *n* (%)
Location of residence	Abu Dhabi	554 (19.4)	897 (25.6)	1451 (22.8)
	Al Ain	617 (21.6)	558 (15.9)	1175 (18.5)
	Western	233 (8.2)	143 (4.1)	376 (5.9)
	Ajman	97 (3.5)	159 (4.6)	256 (4.1)
	Dubai	330 (11.8)	254 (7.3)	584 (9.3)
	Fujairah	195 (7.0)	315 (9.1)	510 (8.2)
	RAK	301 (10.8)	350 (10.1)	651 (10.5)
	Sharjah	480 (17.2)	667 (19.3)	1147 (18.4)
	UAQ	14 (0.5)	132 (3.8)	146 (2.3)

Reside near industrial plants/gas stations/construction sites	Yes	378 (16.5)	417 (14.5)	795 (15.4)

Reside in proximity to overhead power lines and/or plants	Yes	311 (14.2)	254 (9.2)	565 (11.4)

Exposed to tobacco smoke at home or with friends	Not at all	1229 (50.3)	668 (12.4)	3246 (60.5)
	Occasionally	846 (34.6)	606 (20.7)	1452 (27.1)
	Daily	369 (15.1)	299 (10.2)	668 (12.4)

Residence built prior to 1988	Yes	485 (23.7)	470 (20.1)	955 (21.8)

Residential air conditioning	Yes	2388 (98.6)	2937 (99.2)	5399 (99.0)

Residential maintenance (cooling air filtration duct cleaning)	Yearly	593 (27.5)	711 (27.6)	1327 (27.7)
	As needed	1384 (64.1)	1732 (67.3)	3145 (65.7)
	Rarely	182 (8.4)	131 (5.1)	318 (6.6)

Flooring in the main living area of residence	Wall-to-wall carpet	415 (17.9)	486 (16.8)	909 (17.2)
	Ceramic tiles	1478 (63.6)	2009 (69.6)	3533 (67.0)
	Wooden floors	33 (1.4)	26 (0.9)	61 (1.2)
	Heavy rugs	397 (17.1)	364 (12.6)	772 (14.6)

Cooking method	Electricity	105 (4.4)	107 (3.6)	217 (4.0)
	Gas	2251 (94.9)	2827 (95.9)	5141 (95.4)
	Microwave	15 (0.6)	14 (0.5)	33 (0.6)

Residential humidity	Yes	396 (18.6)	473 (17.7)	887 (18.3)

Pets in residence	Yes	547 (22.8)	679 (22.9)	1226 (22.8)

Pests (e.g., cockroaches, rodents) present in residence	No	909 (39.9)	1192 (41.1)	2101 (40.6)
	Yes	365 (16.0)	452 (15.6)	817 (15.8)
	Sometimes	1006 (44.1)	1255 (43.3)	2261 (43.7)

Regular use of pesticides and insecticides at residence	No	659 (29.6)	736 (26.2)	1395 (27.7)
	Yes	1417 (63.7)	1843 (65.5)	3260 (64.7)
	Sometimes	147 (6.6)	234 (8.3)	381 (7.6)

Residential exposure to arts/crafts/ceramics/stain	Yes	406 (18.1)	567 (19.8)	973 (19.0)

Concerned about air pollution in own neighbourhood	Extreme	402 (17.4)	468 (16.2)	870 (16.7)
	Moderate	581 (25.1)	794 (27.4)	1375 (26.4)
	Slight	580 (25.1)	732 (25.3)	1312 (25.2)
	Not at all	750 (32.4)	899 (31.1)	1649 (31.7)

Ever smoked	Yes	779 (28.5)	233 (7.0)	1047 (17.0)

Currently smoking	Yes	624 (24.0)	174 (94.5)	798 (13.9)

Ever used illegal drugs	Yes	68 (2.5)	19 (0.6)	87 (1.5)

Ever used unconventional drugs	Yes	532 (19.7)	1199 (37.3)	1731 (29.3)

Engages in physical activity	Yes	2601 (98.2)	3218 (95.1)	5819 (96.4)

**Table 3:** Associations between socioeconomic and demographic factors and respiratory conditions and symptoms among UAE adolescents (*n* = 6,363).

Variable	Classification	Chronic bronchitis (*n* = 115)	Emphysema (*n* = 28)	Asthma (*n* = 776)	Wheeze (*n* = 740)	Dry cough (*n* = 2,125)
		*n* (%)^a^	*n* (%)^a^	*n* (%)^a^	*n* (%)^a^	*n* (%)^a^
Sex			∗∗	∗∗		∗∗∗
	Female	59 (1.7)	**8 (0.2)**	**387 (11.1)**	405 (11.9)	**1277 (37.7)**
	Male	56 (2.0)	**20 (0.7)**	**389 (13.7)**	335 (12.5)	**848 (31.1)**

School				∗∗∗	∗∗∗	
	Public	68 (1.8)	19 (0.5)	**536 (13.9)**	**399 (10.8)**	1273 (34.1)
	Private	47 (1.9)	9 (0.4)	**240 (9.8)**	**342 (14.3)**	852 (35.9)

Born in UAE				∗∗∗		
	No	29 (1.9)	8 (0.5)	**142 (9.3)**	187 (12.5)	475 (32.0)
	Yes	79 (1.7)	16 (0.3)	**615 (13.1)**	528 (11.8)	1607 (35.5)

Ethnicity				∗∗∗		
	UAE	60 (2.0)	18 (0.6)	**424 (13.9)**	341 (11.7)	1045 (35.4)
	GCC	9 (2.6)	2 (0.6)	**56 (15.7)**	31 (9.6)	118 (35.1)
	Arab/Middle East	20 (2.1)	4 (0.4)	**106 (10.9)**	119 (12.5)	328 (34.7)
	Arab/Africa	10 (1.6)	1 (0.2)	**81 (13.0)**	77 (12.7)	215 (35.3)
	South East Asia	9 (0.9)	0 (0.0)	**74 (7.1)**	134 (1.1)	332 (32.8)
	Western	5 (6.3)	1 (1.3)	**11 (13.6)**	10 (12.5)	27 (35.1)
	None/ other	0 (0.0)	1 (1.3)	**7 (9.1)**	8 (11.4)	22 (30.1)

Father graduated from high school						∗
	No	42 (2.0)	12 (0.6)	249 (11.6)	218 (10.7)	**751 (36.4)**
	Yes	50 (1.7)	9 (0.3)	353 (12.0)	343 (12.0)	**941 (32.9)**

Mother graduated from high school						∗
	No	47 (1.8)	13 (0.5)	314 (12.1)	277 (11.2)	**901 (35.9)**
	Yes	43 (1.7)	9 (0.4)	295 (11.8)	294 (12.0)	**806 (33.0)**

Monthly household income (AED)	<2 K	1 (0.6)	3 (1.9)	21 (13.0)	24 (15.3)	48 (31.2)
	>2 and ≤5 K	14 (1.6)	5 (0.6)	115 (12.9)	100 (11.5)	293 (33.9)
	>5 and ≤8 K	9 (1.4)	2 (0.3)	70 (0.3)	70 (10.8)	229 (34.8)
	>8 and ≤10 K	8 (1.7)	2 (0.4)	46 (9.8)	46 (10.2)	169 (36.7)
	>10 and ≤12 K	6 (1.7)	0 (0.0)	40 (11.4)	40 (11.8)	118 (34.8)
	>12 and ≤15 K	9 (2.9)	2 (0.6)	43 (13.5)	40 (12.9)	96 (30.8)
	>15 and ≤20 K	2 (0.7)	0 (0.0)	31 (10.8)	31 (11.1)	106 (37.3)
	>20 K	19 (2.7)	2 (0.3)	93 (13.1)	95 (14.1)	256 (37.3)

Residential property tenure					∗∗	
	Own	48 (1.8)	12 (0.5)	325 (12.1)	**282 (11.0)**	897 (34.7)
	Rent	40 (1.6)	7 (0.3)	314 (12.2)	**309 (12.4)**	855 (34.3)

Residential crowding	No	19 (1.4)	3 (0.2)	162 (12.1)	132 (10.1)	441 (33.8)
	Yes	26 (1.3)	8 (0.4)	230 (11.2)	198 (10.0)	670 (33.6)

^a^Prevalence %; ^∗^ *p* < 0.05; ^∗∗^ *p* < 0.01; ^∗∗∗^ *p* < 0.001. Note: *n* may not add up due to missing data.

**Table 4:** Associations between environmental and behavioural factors and respiratory conditions and symptoms among UAE adolescents (*n* = 6,363).

Variable	Classification	Chronic bronchitis (*n* = 115)	Emphysema (*n* = 28)	Asthma (*n* = 776)	Wheeze (*n* = 740)	Dry cough (*n* = 2,125)
		*n* (%)^a^	*n* (%)^a^	*n* (%)^a^	*n* (%)^a^	*n* (%)^a^
Location of residence				∗∗∗		∗∗
	Abu Dhabi city	29 (2.0)	4 (0.3)	**244 (16.9)**	185 (13.1)	**531 (38.1)**
	Al Ain	21 (1.8)	4 (0.3)	**131 (11.2)**	146 (12.9)	**391 (34.7)**
	Western	10 (3.1)	1 (0.3)	**47 (12.7)**	38 (11.2)	**124 (34.3)**
	Ajman	3 (1.1)	2 (0.8)	**25 (9.5)**	31 (12.2)	**86 (34.4)**
	Dubai	10 (1.8)	3 (0.5)	**50 (8.9)**	58 (10.7)	**192 (33.4)**
	Fujairah	8 (1.5)	1 (0.2)	**31 (6.0)**	47 (9.3)	**169 (33.7)**
	RAK	9 (1.3)	4 (0.6)	**27 (4.0)**	67 (10.4)	**195 (30.4)**
	Sharjah	21 (8.1)	8 (0.7)	**103 (8.9)**	148 (13.1)	**370 (33.0)**
	UAQ	4 (2.7)	1 (0.7)	**11 (7.5)**	21 (14.8)	**67 (48.2)**

Reside in proximity to industrial plants/gas stations/construction			∗∗∗	∗∗∗	∗∗∗	
	No	66 (1.5)	**11 (0.3)**	**480 (10.9)**	**436 (10.2)**	1445 (33.7)
	Yes	14 (1.8)	**10 (1.3)**	**131 (16.2)**	**130 (17.0)**	281 (35.9)

Reside in proximity to overhead power lines and/or plants			**∗** **∗** **∗**			
	No	67 (1.5)	**11 (0.3)**	512 (11.6)	463 (10.8)	1450 (33.6)
	Yes	7 (1.3)	**9 (1.6)**	83 (14.6)	73 (13.5)	205 (37.4)

Exposed to tobacco smoke at home or with friends		∗		∗∗∗	∗∗∗	∗∗∗
	Not at all	**51 (1.6)**	13 (0.4)	364 (11.1)	309 (9.7)	995 (31.2)
	Occasionally	**31 (2.2)**	7 (0.5)	187 (12.8)	210 (15.0)	548 (38.6)
	Daily	**20 (3.0)**	2 (0.3)	120 (17.7)	127 (19.7)	284 (43.3)

Residence built prior to 1988	No	60 (1.8)	13 (0.4)	431 (12.4)	392 (11.8)	1148 (34.2)
	Yes	20 (2.1)	3 (0.3)	118 (12.2)	101 (10.9)	321 (34.4)

Residential air conditioning	No	1 (1.8)	0 (0.0)	4 (7.0)	10 (19.2)	15 (28.8)
	Yes	89 (1.7)	21 (0.4)	646 (12.0)	587 (11.3)	1782 (34.2)

Residential maintenance (air filter/cleaning)	Yearly	22 (1.7)	8 (0.6)	154 (11.6)	138 (10.9)	427 (33.7)
	As needed	54 (1.8)	10 (0.3)	372 (11.9)	342 (11.2)	1043 (33.9)
	Rarely	4 (1.3)	2 (0.7)	47 (15.1)	39 (13.0)	113 (37.5)

Flooring in the main living area of residence	Wall-to-wall carpet	16 (1.8)	3 (0.3)	98 (10.8)	94 (10.7)	298 (34.0)
	Ceramic tiles	57 (1.6)	10 (0.3)	435 (12.4)	390 (11.5)	1182 (34.5)
	Wooden floors	2 (3.4)	3 (5.2)	6 (10.0)	12 (22.6)	21 (36.2)
	Heavy rugs	12 (1.6)	4 (0.5)	97 (12.6)	83 (11.4)	240 (32.5)

Cooking method					∗∗	
	Electricity	6 (2.9)	5 (2.5)	31 (14.4)	**37 (18.0)**	75 (35.4)
	Gas	79 (1.6)	16 (0.3)	603 (11.8)	**543 (11.0)**	1697 (34.2)
	Microwave	0 (0)	0 (0)	4 (12.9)	**5 (16.1)**	8 (25.8)

Residential humidity					∗∗	
	No	62 (1.6)	12 (0.3)	450 (11.4)	**412 (10.8)**	1282 (33.4)
	Yes	15 (1.7)	6 (0.7)	119 (13.5)	**120 (14.1)**	314 (36.8)

Pets in residence			∗	∗∗		
	No	63 (1.5)	**12 (0.3)**	**469 (11.2)**	437 (10.8)	1361 (33.6)
	Yes	24 (2)	**9 (0.7)**	**179 (14.4)**	150 (12.7)	434 (36)

Pests (e.g., cockroaches, rodents) present in residence			∗			∗∗∗
	No	31 (1.5)	**5 (0.2)**	242 (11.4)	220 (10.8)	**627 (30.5)**
	Yes	15 (1.9)	**7 (0.9)**	111 (13.4)	108 (13.7)	**305 (38.4)**
	Sometimes	33 (1.5)	**7 (0.3)**	259 (11.4)	238 (10.8)	**792 (35.7)**

Regular use of pesticides/insecticides at residence						∗
	No	24 (1.7)	3 (0.2)	154 (10.9)	154 (11.4)	**427 (31.1)**
	Yes	55 (1.7)	13 (0.4)	417 (12.7)	345 (10.9)	**1133 (35.6)**
	Sometimes	2 (0.5)	0 (0.0)	37 (9.7)	51 (13.7)	**131 (35.3)**

Exposure to arts/crafts/ceramics/stain		∗∗	∗∗		∗	
	No	**55 (1.3)**	**10 (0.2)**	497 (11.9)	**438 (10.9)**	1360 (33.6)
	Yes	**26 (2.7)**	**9 (0.9)**	123 (12.5)	**132 (13.8)**	351 (36.7)

Concerned about air pollution in own neighbourhood					∗∗	∗
	Extreme	13 (1.5)	5 (0.6)	117 (13.1)	**125 (14.6)**	**299 (34.9)**
	Moderate	18 (1.3)	7 (0.5)	161 (11.6)	**155 (11.6)**	**492 (36.5)**
	Slight	26 (2.0)	4 (0.3)	150 (11.4)	**135 (10.6)**	**445 (34.8)**
	Not at all	27 (1.6)	6 (0.4)	203 (12.2)	**159 (10.0)**	**506 (31.3)**

Ever smoked		∗∗∗	∗∗∗	∗∗∗	∗∗∗	∗∗∗
	No	80 (1.6)	16 (0.3)	579 (11.3)	516 (10.4)	1667 (33.7)
	Yes	33 (3.3)	12 (1.2)	177 (17.1)	203 (20.7)	398 (39.8)

Currently smoking		∗∗	∗∗∗	∗∗	∗∗∗	
	No	80 (1.6)	15 (0.3)	583 (11.7)	544 (11.2)	1664 (34.3)
	Yes	25 (3.2)	10 (1.3)	125 (15.4)	135 (18.0)	290 (37.4)

Ever used illegal drugs		∗∗	∗∗∗		∗∗∗	
	No	**108 (1.9)**	**20 (0.3)**	725 (12.3)	**686 (12.0)**	1996 (34.8)
	Yes	**6 (6.9)**	**6 (6.8)**	17 (18.1)	**25 (28.4)**	35 (39.8)

Purposely smelled gasoline fumes/glue/correctors/car exhaust/burning black ants (no)			∗		∗∗∗	∗∗∗
	No	84 (2.0)	23 (0.6)	525 (12.4)	**462 (11.4)**	**1295 (31.6)**
	Yes	29 (1.7)	3 (0.2)	218 (12.4)	**251 (14.8)**	**733 (43.1)**

Engages in physical activity		∗				
	No	**8 (3.7)**	4 (1.8)	28 (12.8)	17 (8.1)	64 (31.8)
	Yes	**97 (1.7)**	21 (0.4)	711 (12.1)	669 (12.3)	1990 (34.9)

^a^Prevalence %; ^∗^ *p* < 0.05; ^∗∗^ *p* < 0.01; ^∗∗∗^ *p* < 0.001. Note: *n* may not add up due to missing data.

**Table 5:** Predictors of respiratory conditions and symptoms among adolescents in the United Arab Emirates (UAE).

	Reference	Chronic bronchitis		Emphysema		Asthma		Wheeze		Dry cough	
		OR	95% CI	OR	95% CI	OR	95% CI	OR	95% CI	OR	95% CI
Sex (female)	Male			1.93	0.40–9.32	1.38^∗^	**1.10–1.73**			0.70^∗∗∗^	**0.60–0.81**

Emirate (Fujairah)	Abu Dhabi City					2.63^∗∗∗^	**1.66–4.17**			1.22	0.90–1.67
	Al Ain					**1.21**	**0.73–1.99**			1.00	0.72–1.38
	Western					1.82^∗^	**1.03–3.22**			1.11	0.76–1.63
	Ajman					**1.75**	**0.91–3.48**			1.22	0.78–1.93
	Dubai					**1.25**	**0.73–2.14**			1.01	0.71–1.43
	UAQ					**1.61**	**0.69–3.78**			1.44	0.81–2.56
	RAK					**1.08**	**0.63–1.86**			0.84	0.59–1.20
	Sharjah					**1.37**	**0.83–2.25**			0.94	0.67–1.31

Reside near industry/gas stations/construction (no)	Yes			1.32	0.27–6.34	1.65^∗^	**1.26–2.16**	1.24	0.93–1.65		

Reside close to overhead power lines/plants (no)	Yes			**3.71**	**0.83–16.62**	1.02	0.74–1.40				

Exposed to tobacco (not at all)	Occasionally	1.03	0.57–1.84			**1.04**	**0.82–1.32**	1.43^∗∗^	**1.11–1.84**	1.45^∗∗∗^	**1.22–1.72**
	Daily	1.13	0.53–2.43			1.37^∗^	**1.01–1.88**	1.54^∗^	**1.10–2.15**	1.51^∗∗∗^	**1.20–1.91**

Cooking methods (microwave)	Electricity							1.56	0.33–7.45		
	Gas							1.00	0.23–4.49		

Residential humidity (no)	Yes							1.33^∗^	**1.01–1.75**		

Pests (e.g., cockroaches, rodents) present in residence (no)	Yes			2.07	0.38–11.39					1.23^∗∗^	**1.15–1.53**
	Sometimes			0.75	0.14–4.14					1.09^∗∗^	**1.05–1.40**

Pets in residence (no)	Yes			1.98	0.49–8.00	1.23	0.97–1.56				

Regular use of insecticide/pesticide (no)	Sometimes/yes									1.14	0.96–1.35

Exposure to arts/crafts/ceramics/stain (no)	Yes	2.15^**∗**^	**1.26–3.66**	4.66^∗^	**1.14–18.96**			1.18	0.90–1.54		

Concerned about air pollution (not at all)	Extremely							1.50^∗^	**1.08–2.08**	1.26^∗^	**1.01–1.57**
	Moderately							**1.17**	**0.87–1.58**	1.39^∗∗^	**1.15–1.69**
	Slightly							**1.02**	**0.75–1.39**	**1.08**	**0.88–1.31**

Ever smoked (no)	Yes	1.39	0.68–2.85	1.35	0.28–6.62	1.13	0.83–1.54	1.9^∗∗∗^	**1.39–2.59**	1.31^∗^	**1.06–1.63**

Currently smoking (no)	Yes	0.82	0.36–1.89	0.74	0.11–5.05	1.06	0.76–1.47	1.00	0.70–1.41		

Ever used illegal drugs (no)	Yes	1.23	0.15–9.74	3.99	0.23–7.81			1.28	0.56–2.95		

Purposely smelled gasoline fumes, glue, correctors, car exhaust, or burning black ants (no)	Yes			0.22	0.02–2.05			1.46^∗∗^	**1.15–1.85**	1.45^∗∗∗^	**1.23–1.70**

Engages in physical activity (no)	Yes	0.63	0.19–2.07								

School (private)	Public					1.26	0.92–1.72	0.94	0.72–1.23		

Ethnicity (South East Asia)		∗									
	UAE	4.99^∗∗^	**1.52–16.44**			1.66^∗^	**1.06–2.60**				
	GCC	5.61^∗^	**1.32–23.82**			1.76^∗^	**1.01–3.06**				
	Arab/Middle East	4.45^∗^	**1.22–16.26**			**1.37**	**0.89–2.11**				
	Arab/North Africa	1.30^∗^	**1.10–16.90**			1.68^∗^	**1.06–2.66**				
	Western	15.92^∗^	**3.05–83.07**			2.36^∗^	**1.02–5.50**				
	Other	∧				**1.85**	**0.73–4.66**				

UAE born (no)	Yes					1.22	0.93–1.64			1.19	1.00–11.1

Father completed high school (yes)	No									1.10	0.91–1.34

Mother completed high school (yes)	No									1.08	0.89–1.30

Residential property tenure (own)	Rent							1.08	0.83–1.40		

Diagnosis with chronic bronchitis (no)	Yes	NA		20.83^∗∗∗^	4.52–90.91	NA		NA		NA	

Diagnosis with asthma (no)	Yes	NA		2.83	0.65–12.35	NA		NA		NA	

Report wheeze (no)	Yes	NA		6.85^∗^	1.49–31.25	NA		NA		NA	

Report dry cough (no)	Yes	NA		1.11	0.28–4.42	NA		NA		NA	

**Hosmer and Lemeshow Test **(**X** ^2^)		5.49		72.62^∗∗∗^		4.80		3.86		5.64	

^∗^ *p* < 0.05; ^∗∗^ *p* < 0.01; ^∗∗∗^ *p* < 0.001; ^∧^missing due to small sample size.

## Appendix

### Summary of Survey Items Addressing Outcome Measures

Were you ever told by a doctor or health professional that you have any of the following conditions?* Please check all that apply*.

- Chronic bronchitis
- Emphysema
- Asthma
- Other chest conditions;
- Specify —
- Any long-term skin conditions
- Specify —
- Hay fever/other allergies
- Specify —
- Arthritis/rheumatism
- Any respiratory problems
- Specify —
- High blood pressure/hypertension
- Heart disease
- Specify —
- Thalassemia;
- Specify —
- Sickle cell anemia
- Any type of anemia
- Specify —
- Diabetes;
- Specify —
- Kidney problem;
- Specify —
- Ulcer;
- Specify —
- Any type of cancer;
- Specify —
- Migraine headache
- Thyroid condition;
- Specify —
- Any food allergies;
- Specify —
- Other major health diagnoses;
- Specify —

Have you* had wheezing or whistling in the chest* in the last 12 months?

- Yes
- No

In the last 12 months, have you had a dry cough at night, apart from a cough associated with a cold or chest infection?

- Yes
- No

## Abbreviations and Acronyms

COPD: Chronic obstructive pulmonary disease
UAE: United Arab Emirates
UAQ: Umm al-Quain
RAK: Ra's al-Khaymah
NSPHUAE: National Study of Population Health in the UAE
ISAAC: International Study of Asthma and Allergies in Children
WHO: World Health Organization
SPSS: Statistical Package for the Social Sciences.
